# Development and validation of a novel staging system integrating the number and location of lymph nodes for gastric adenocarcinoma

**DOI:** 10.1038/s41416-020-01190-z

**Published:** 2020-12-02

**Authors:** Ziyu Li, Xiaolong Wu, Xiangyu Gao, Fei Shan, Xiangji Ying, Yan Zhang, Jiafu Ji

**Affiliations:** Gastrointestinal Cancer Center, Key Laboratory of Carcinogenesis and Translational Research (Ministry of Education), Peking University Cancer Hospital and Institute, Beijing, People’s Republic of China

**Keywords:** Surgical oncology, Cancer models

## Abstract

**Background:**

Evidence suggests that the anatomic extent of metastatic lymph nodes (MLNs) affects prognosis, as proposed by alternative staging systems. The aim of this study was to establish a new staging system based on the number of perigastric (PMLN) and extra-perigastric (EMLN) MLNs.

**Methods:**

Data from a Chinese cohort of 1090 patients who had undergone curative gastrectomy with D2 or D2 plus lymphadenectomy for gastric cancer were retrospectively analysed. A Japanese validation cohort (*n* = 826) was included. Based on the Cox proportional hazards model, the regression coefficients of PMLN and EMLN were used to calculate modified MLN (MMLN). Prognostic performance of the staging systems was evaluated.

**Results:**

PMLN and EMLN were independent prognostic factors in multivariate analysis (coefficients: 0.044, 0.115; all *P* < 0.001). MMLN was calculated as follows: *MMLN* = *PMLN* + 2.6 × *EMLN*. The MMLN staging system showed superior prognostic performance (C-index: 0.751 in the Chinese cohort; 0.748 in the Japanese cohort) compared with the five published LN staging systems when MMLN numbers were grouped as follows: MMLN0 (0), MMLN1 (1–4), MMLN2 (5–8), MMLN3 (9–20), and MMLN4 (>20).

**Discussion:**

The MMLN staging system is suitable for assessing overall survival among patients undergoing curative gastrectomy with D2 or D2 plus lymphadenectomy.

## Background

Comprehensive and appropriate therapeutics, including endoscopy, surgery, radiotherapy, chemotherapy, and immunotherapy, have improved outcomes of gastric cancer patients.^1–5^ Surgery remains vital in the treatment of resectable, non-metastatic gastric cancer.^6^ Tumour invasion depth and lymph node (LN) status—used in almost all gastric cancer staging systems—are essential independent prognostic factors for overall survival (OS), following a microscopically margin-negative (R0) resection.^7–10^

LN classification in the eighth edition of the tumour-node-metastasis (TNM) staging system of the American Joint Committee on Cancer/International Union Against Cancer (AJCC/UICC) and the 15th edition of the Japanese Gastric Cancer Association (JGCA) staging system, the world’s most commonly used staging systems, is based on the number of metastatic LNs (MLNs).^10,11^ However, before the fifth edition of TNM and 14th edition of the JGCA staging systems, LN classification was based on the anatomical location of MLNs.^12,13^ The numeric LN staging system with multiple updates to the cut-off value showed high accuracy in survival prediction; however, some suggested limitations included lack of information on the anatomical extent of MLNs and the total number of LNs (TLNs) retrieved during surgery.^14–17^ Son et al.^14^ proposed inclusion of the anatomic extent of MLNs in a staging system for more accurately predicting gastric cancer prognosis. Choi et al.^18^ developed an alternative LN staging system based on anatomical location, which reclassified the LN stations into lesser-curvature (LC), greater-curvature (GC), and extra-perigastric (EP) groups. Chen et al.^19^ developed yet another LN staging system based on both the number and anatomic location of metastatic LNs, considered a more efficient prognostic indicator than the JGCA and TNM staging systems. Other authors have proposed LN ratio (LNR), the ratio of metastatic LNs relative to the total number of retrieved LNs, and log odds of metastatic LNs (LODDS), defined as the log of the ratio between the probability of being a positive LN and the probability of being a negative LN, which might be better LN staging systems, as they take into account the number of LNs retrieved during surgery.^20–23^

The National Comprehensive Cancer Network (NCCN) guidelines recommend D2 gastrectomy, which includes systematic lymphadenectomy of N1 and N2 group LNs, as the standard treatment for advanced gastric cancer.^24^ In many high-volume centres in the Eastern Asian countries, D2 gastrectomy is performed routinely, and has been shown to improve survival.^25–27^ Considering the impact of MLNs’ anatomic location on survival, given D2 surgery outcomes, we hypothesised that the number of LNs in the perigastric (LN stations No. 1, 2, 3, 4, 5, 6) and EP areas (LN stations No. 7, 8, 9, 10, 11, 12, etc.) might differently influence the prognosis. In other words, the effect of each extra-perigastric MLN (EMLN) on prognosis might be different from that of each perigastric MLN (PMLN). Therefore, this study aimed to define the different prognostic effects of PMLN and EMLN and develop and validate a new staging system based on the number of PMLN and EMLN. Comparisons were made with the eighth edition AJCC LN, Choi’s,^18^ Chen’s,^19^ LNR, and LODDS staging systems to confirm the prognostic value of the new staging system.

## Methods

### Patient selection

**Fig. 1:**
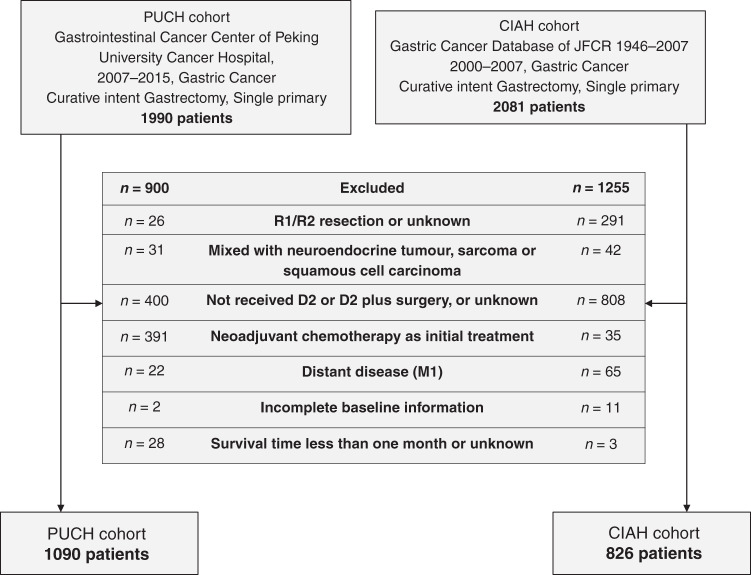
Flow chart of patients selection. PUCH Peking University Cancer Hospital, CIAH Cancer Institute Ariake Hospital, JFCR The Japanese Foundation for Cancer Research.

Patients who had undergone curative-intent resection for gastric cancer at the Department of Gastrointestinal Tumor Center at Peking University Cancer Hospital (PUCH) between January 1, 2007 and December 31, 2015 were selected. Inclusion criteria were a histopathological diagnosis of gastric adenocarcinoma with no combined malignant neoplasm or distant metastasis and treatment with R0 gastrectomy and D2 or D2 plus lymphadenectomy. Patients with remnant gastric cancer, preoperative chemotherapy, incomplete clinicopathological or follow-up information, and less than 1-month postoperative survival were excluded. According to the JGCA guidelines,^28^ D2 lymphadenectomy cannot be performed during proximal gastrectomy; hence, patients treated with proximal gastrectomy were excluded. A total of 1090 eligible patients were included (Fig. 1).

The validation cohort included 826 patients treated between 2000 and 2007 at the Cancer Institute Ariake Hospital (CIAH), Tokyo, Japan, that met the aforementioned inclusion and exclusion criteria (Fig. 1). The data for the CIAH cohort contained no unique personal identifiers and were extracted from a publicly accessible database.^29^

### Clinicopathological data

The clinicopathological dataset included patient demographics (age, sex), pathological variables (location, size, histological type, differentiation, invasion depth, LN status, Lauren type, vascular invasion), follow-up duration, and survival status at last follow-up (April 2019).

LN status included the anatomic location of each MLN and the number of TLNs. All patients in PUCH cohort were treated by the same team of experienced surgeons. With reference to the Japanese gastric cancer treatment guidelines, all the surgeons have reached a consensus on the surgical procedures and extent of lymphadenectomy to ensure the stability of the surgical outcomes. After radical gastrectomy with D2 or D2-plus-lymphadenectomy per the Japanese gastric cancer treatment guidelines, the surgeons dissected the LNs from the gastrectomy specimen. All LNs were placed in containers marked with numbers corresponding to the numerical system for LN identification described by the Japanese Research Society for Gastric Cancer (JRSGC).^30^ Two or more trained pathologists used palpation to examine the specimens, obtain as many LNs as possible, regardless of the size of LNs, and reported the number of TLNs and MLNs in each station. Then we counted the number of all MLNs, TLNs, and divided the MLNs into PMLNs and EMLNs.

To facilitate comparisons with other LN staging systems, we recorded the following data. PMLNs were divided into LC and GC groups, as proposed by Choi et al., and patients were classified into Choi’s new N1 (LC alone, GC alone, EP alone), Choi’s new N2 (LC + GC, LC + EP, GC + EP), and Choi’s new N3 (LC + GC + EP) groups. According to the LN groups described by JRSGC and cut-off values of MLN in each group proposed by Chen et al., patients were classified into Chen’s new N1 (number of N1 group MLNs 1–6), Chen’s new N2 (number of N2 group MLNs 1–6), and Chen’s new N3 (number of N1 group MLNs ≥7 or N2 group MLNs ≥7 or the presence of N3 group MLNs). LNR and LODDS were calculated and grouped using the cut-off values proposed by Wang et al.^31^ and Sun et al.,^21^ respectively.

### Statistical analysis

The clinicopathological characteristics of the study cohorts were presented as mean (standard deviation) for continuous variables and counts and proportions for categorical variables. Univariate and multivariate Cox proportional hazards regression models were used to analyse the relationship between the clinicopathological variables and OS. Variables statistically significant (*P* < 0.05) in the univariate analysis were included in multivariate analysis (stepwise backward elimination). In the results of multivariate analysis, the regression coefficient can reflect the influence of the variable on the prognosis, and the regression coefficients of PMLNs and EMLNs were extracted from the multivariate Cox model of the PUCH cohort. The ratio of the regression coefficient of PMLNs and EMLNs can reflect their different prognostic effects, and we used this ratio to adjust EMLNs. Then the number of modified metastatic LNs (MMLNs) was calculated using the following formula: ${\mathrm{{MMLN}}} = {\mathrm{{PMLN}}} + \frac{{\beta _{{\mathrm{{EMLN}}}}}}{{\beta _{{\mathrm{{PMLN}}}}}} \times {\mathrm{{EMLN}}}$, where *β*_PMLN_ and *β*_EMLN_ were the regression coefficients or PMLNs and EMLNs in the multivariate Cox model. MMLN was considered to be a continuous variable. To group the MMLN, first, the number of MMLNs was rounded to an integer; then four cut-off points were set to divide the MMLNs into five groups consistent with those of the eighth edition TNM LN categories. Further, we used the enumeration method for different combinations of cut-off points in Cox regression models to calculate Harrell’s concordance index (C-index), often used to evaluate the discriminative ability of a model. A high C-index represents a better discrimination ability of the model. We selected the cut-off points of maximum C-index to construct our MMLN staging system.

The Kaplan−Meier method with the log-rank test was used to explore differences in survival between the strata established by the eighth edition AJCC LN, Choi’s, Chen’s, LNR, LODDS, and our MMLN staging system. Comparisons of the predictive value of each LN staging system were performed using the C-index, the likelihood-ratio test, and Akaike’s Information Criterion (AIC). A model with a low AIC, a high likelihood-ratio *χ*^2^ score, and a high C-index had a better predictive value. All analyses were performed using the R software (version 3.6.0; https://www.r-project.org/). Statistical significance was set at a two-sided *P* value < 0.05.

## Results

### Clinicopathological characteristics

The clinicopathological characteristics of the PUCH (*n* = 1090) and the CIAH (*n* = 826) cohort, with median follow-up duration of 49.2 (range 1–136) and 30.0 (1–95) months, respectively, are presented in Table 1. The sex ratio was similar between the two cohorts (*P* = 0.751), but other variables showed significant differences. Patients in the CIAH cohort were significantly younger, with earlier-stage gastric cancer, specifically, a higher proportion of cases at T1 stage (41.6% vs. 11.2%, *P* < 0.001) and N0 stage (53.5% vs. 31.8%, *P* < 0.001), and a greater number of TLNs (41.0 ± 15.6 vs. 33.1 ± 11.7, *P* < 0.001) than the corresponding values in the PUCH cohort. There were differences between the PUCH and CIAH cohort in the distribution of the LN staging systems (all *P* < 0.05). For example, in the CIAH cohort, no patients fell under LNR > 0.7 and only two patients were classified under LODDS > 0.

**Table 1:** Patient characteristics.

Variable	PUCH cohort (*n* = 1090)	CIAH cohort (*n* = 826)	*P* value
Age (years)	62.3 (11.8)	61.1 (11.0)	0.019
Sex			0.751
Female	328 (30.1)	255 (30.9)	
Male	762 (69.9)	571 (69.1)	
Tumour size (cm)	4.5 ± 3.0	5.5 ± 4.1	<0.001
Tumour location			<0.001
U	205 (18.8)	175 (21.2)	
M	239 (21.9)	388 (47.0)	
L	584 (53.6)	239 (28.9)	
Overlapping lesion	62 (5.7)	24 (2.9)	
Extent of gastrectomy			<0.001
Distal	630 (57.8)	578 (70.0)	
Total	460 (42.2)	248 (30.0)	
Differentiation			<0.001
Differentiated	520 (47.7)	310 (37.5)	
Undifferentiated	570 (52.3)	516 (62.5)	
Histological type			<0.001
Adenocarcinoma	795 (72.9)	573 (69.4)	
Signet ring cell carcinoma	234 (21.5)	232 (28.1)	
Mucinous adenocarcinoma	61 (5.6)	21 (2.5)	
AJCC 8th edition pT stage			<0.001
T1	122 (11.2)	344 (41.6)	
T2	195 (17.9)	124 (15.0)	
T3	294 (27.0)	157 (19.0)	
T4a	442 (40.6)	179 (21.7)	
T4b	37 (3.4)	22 (2.7)	
MLNs	5.7 (7.9)	2.1 (3.6)	<0.001
PMLNs	4.9 (6.8)	1.7 (3.1)	<0.001
EMLNs	0.8 (2.0)	0.4 (0.9)	<0.001
TLNs	33.1 (11.7)	41.0 (15.6)	<0.001
LODDS	−0.97 (0.71)	−1.47 (0.50)	<0.001
LNR	0.17 (0.21)	0.05 (0.09)	<0.001
AJCC 8th edition pN stage			<0.001
N0	347 (31.8)	442 (53.5)	
N1	211 (19.4)	167 (20.2)	
N2	196 (18.0)	141 (17.1)	
N3a	216 (19.8)	63 (7.6)	
N3b	120 (11.0)	13 (1.6)	
LODDS stage			<0.001
≤−1.5	357 (32.8)	466 (56.4)	
−1.5 to −1.0	184 (16.9)	194 (23.5)	
−1.0 to −0.5	236 (21.7)	125 (15.1)	
−0.5 to 0	210 (19.3)	39 (4.7)	
>0	103 (9.4)	2 (0.2)	
LNR stage			<0.001
0	347 (31.8)	442 (53.5)	
0–0.07	177 (16.2)	198 (24.0)	
0.07–0.3	326 (29.9)	160 (19.4)	
0.3–0.7	204 (18.7)	26 (3.1)	
>0.7	36 (3.3)	0	
Choi’s LN stage			<0.001
N0	347 (31.8)	442 (53.5)	
N1	258 (23.7)	180 (21.8)	
N2	254 (23.3)	144 (17.4)	
N3	231 (21.2)	60 (7.3)	
Chen’s LN stage		<0.001	
N0	347 (31.8)	442 (53.5)	
N1	312 (28.6)	193 (23.4)	
N2	135 (12.4)	129 (15.6)	
N3	296 (27.2)	62 (7.5)	

Data are presented as *n* (%) or mean (SD).

*PUCH* Peking University Cancer Hospital, *CIAH* Cancer Institute Ariake Hospital, *AJCC* American Joint Committee on Cancer, *MLN* metastatic lymph node, *PMLN* perigastric metastatic lymph node, *EMLN* extra-perigastric metastatic lymph node, *TLN* total number of lymph nodes retrieved, *LODDS* log odds of metastatic lymph nodes, *LNR* lymph node ratio.

### Prognostic factors in multivariate analysis and development of the MMLN staging system

Univariate and multivariate analyses were performed on the PUCH cohort (Table 2). In the univariate analysis, both PMLNs and EMLNs significantly affected OS (all *P* < 0.05). In the multivariate analysis, the extent of gastrectomy, pT stage, TLNs, PMLNs, and EMLNs were independent prognostic factors. We extracted the regression coefficients of PMLNs (*β*_PMLN_ = 0.044) and EMLNs (*β*_EMLN_ = 0.115) in Table 2, and then calculated the ratio of *β*_EMLN_ and *β*_PMLN_ to 2.6, and established the formula for MMLNs as follows: MMLN = PMLN + 2.6 × EMLN.

**Table 2:** Univariate and multivariate analysis using Cox proportional hazard regression model in PUCH cohort.

	Univariate	Multivariate			
Variable	*P* value	Coefficients	HR	95% CI	*P* value
Tumour size (cm)					
≤5 (reference)	<0.001				
>5					
Extent of gastrectomy					
Distal (reference)	<0.001	0.323	1.00		0.020
Total			1.35	1.05–1.74	
Differentiation					
Differentiated (reference)	0.010				
Undifferentiated					
Histological type					
Adenocarcinoma (reference)	0.026				
Signet ring cell carcinoma					
Mucinous adenocarcinoma					
AJCC pT stage					
T1 (reference)	<0.001	0.593	1.00		<0.001
T2			3.78	1.12–12.80	
T3			8.70	2.72–27.87	
T4a			12.92	4.08–40.86	
T4b			28.59	8.50–96.15	
TLNs					
≤30 (reference)	0.005	−0.008			0.028
>30			0.74	0.56–0.97	
PMLNs	<0.001	0.044	1.04	1.03–1.06	<0.001
EMLNs	<0.001	0.115	1.12	1.08–1.16	<0.001

*AJCC* American Joint Committee on Cancer, *TLN* total number of lymph nodes retrieved, *PMLN* perigastric metastatic lymph node, *EMLN* extra-perigastric metastatic lymph node, *CI* confidence intervals.

The enumeration method was used to establish the optimal stratification of MMLNs. Finally, an MMLN staging system with MMLN0 (MMLNs = 0), MMLN1 (MMLNs = 1–4), MMLN2 (MMLNs = 5–8), MMLN3 (MMLNs = 9–20), and MMLN4 (MMLNs > 20), which had the best discriminative ability (C-index = 0.747), was defined. The top ten stratifications with the highest C-index values are listed in Supplementary Table S1. The MMLN staging system was further examined, and univariate analysis and multivariate analysis confirmed that the MMLN staging system was an independent prognostic factor in the validation cohort (*P* < 0.001, Supplementary Table S2).

### Survival analysis based on the MMLN classification within the other five LN classifications

**Fig. 2:**
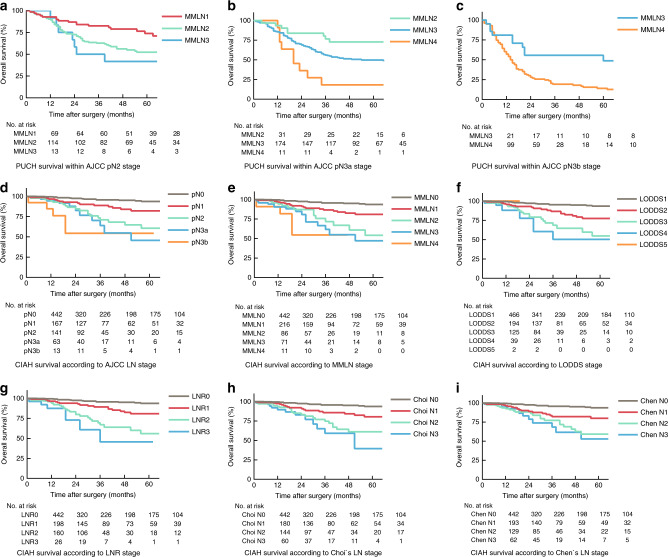
Kaplan–Meier survival curves showing overall survival probabilities (log-rank test). For PUCH cohort, MMLN staging system showed good discriminative ability within **a** pN2 stage (*P* = 0.008), **b** pN3a stage (*P* = 0.003), **c** pN3b stage (*P* = 0.003) of the American Joint Committee on Cancer (AJCC), 8th edition, staging system. For CIAH cohort, all **d** AJCC, **e** MMLN staging system, **f** log odds of positive lymph nodes (LODDS) stage, **g** lymph node ratio (LNR) stage, **h** Choi’s LN stage, **i** Chen’s LN stage showed good prognostic performance (all *P* < 0.001). However, the last two or three subgroups within each of the six staging systems showed similar survival curves, **d** pN2 vs. pN3a, *P* = 0.532; pN3a vs. pN3b, *P* = 0.300; **e** MMLN2 vs. MMLN3, *P* = 0.361; MMLN3 vs. MMLN4, *P* = 0.320; **f** LODDS3 vs. LODDS4, *P* = 0.361; LODDS4 vs. LODDS5, *P* = 0.539; **g** LNR2 vs. LNR3, *P* = 0.225; **h** Choi N2 vs. Choi N3, *P* = 0.299; **i** Chen N2 vs. Chen N3, *P* = 0.649.

The Kaplan−Meier survival curves for the PUCH cohort stratified into the eighth edition AJCC LN system-based subgroups are plotted in Fig. 2a–c. As pN0 is equivalent to MMLN0 and only two patients of MMLN2 were included in the pN1 stage, the survival curves of pN0 and pN1 stages are not shown. Within each pN stage, significantly different survival rates are shown for each of the MMLN categories (log-rank test: pN2, *P* = 0.008; pN3a, *P* = 0.003; pN3b, *P* = 0.003). Results of the survival analysis for the MMLN classification-based strata within the AJCC LN, LNR, LODDS, Choi’s, and Chen’s staging system are shown in Table 3. The MMLN staging system was able to distinguish groups associated with different OS within most of the subgroups distinguished by each of these previously proposed staging systems. In contrast, the previously proposed LN-based staging systems did not distinguish differences in OS within the groups based on MMLN staging system. The corresponding survival curves are shown in Supplementary Fig. S1.

**Table 3:** Overall survival rates based on AJCC LN stage, LNR stage, LODDS stage, Choi’s LN stage, and Chen’s LN stage according to the MMLN classification.

	MMLN0		MMLN1		MMLN2		MMLN3		MMLN4		*P* value
	No.	5-yrs (%)	No.	5-yrs (%)	No.	5-yrs (%)	No.	5-yrs (%)	No.	5-yrs (%)	
AJCC LN stage											
N0	347	89.2									—
N1			209	73.2	2	—					0.472
N2			69	73.9	114	52.3	13	41.7			0.008
N3a					31	72.7	174	49.7	11	18.2	0.003
N3b							21	48.7	99	14.1	0.003
*P* value		—		0.957		0.13		0.899		0.375	
LNR stage											
0	347	89.2									—
0–0.07			173	74.4	4	—					0.784
0.07–0.3			104	72.6	133	55.8	88	49.2	1	—	0.001
0.3–0.7			1	—	10	70	116	48.3	77	16	<0.001
>0.7							4	50	32	9.4	0.132
*P* value		—		0.146		0.807		0.969		0.035	
LODDS stage											
≤−1.5	347	89.2	10	63							0.053
−1.5 to −1.0			177	82.2	6	33.3	1	—			<0.001
−1.0 to −0.5			91	71.8	102	60.5	42	48.5	1	—	0.014
−0.5 to 0					39	50.9	138	49.8	33	22.6	0.001
>0							27	45.6	76	10.3	<0.001
*P* value		—		0.805		0.521		0.193		0.06	
Choi’s LN stage											
N0	347	89.2									—
N1			216	75.7	29	65.5	13	69.2			0.231
N2			62	65.1	89	58.1	86	56	17	23.5	0.001
N3					29	42.6	109	40.5	93	12.5	<0.001
*P* value		—		0.085		0.056		0.246		0.371	
Chen’s LN stage											
N0	347	89.2									—
N1			253	72.6	59	55.8					0.013
N2			25	80	57	50	52	40.8	1	—	0.003
N3					31	72.7	156	51.5	109	14.4	<0.001
*P* value		—		0.562		0.134		0.171		0.451	

*5-yrs* 5 years survival rate, *AJCC* American Joint Committee on Cancer, *MMLN* modified metastatic lymph node, *LNR* lymph node ratio, *LODDS* log odds of metastatic lymph nodes.

### Comparisons of the prognostic performance of all LN-based stage systems

Results from Cox regression modelling, C-index values, AIC values, and likelihood-ratio *χ*^2^ scores were compared among the PUCH and CIAH cohort. In the PUCH cohort, the MMLN staging system showed the best prognostic performance (C-statistic: 0.751; AIC: 1026.1.2; likelihood-ratio *χ*^2^ score: 288.8, Table 4). Both the LNR and LODDS classification, based on MLNs and TLNs, were not better than the eighth edition AJCC LN staging system. For Chen’s and Choi’s LN staging systems based on status and anatomic location of MLNs, Chen’s was not better than any other LN staging system; the Choi’s was second only to the MMLN staging system.

**Table 4:** Prognostic performance of different lymph node staging system in PUCH and CIAH cohorts.

	PUCH cohort			CIAH cohort		
	C index	Likelihood-ratio test	AIC	C index	Likelihood-ratio test	AIC
MMLN stage	0.751	288.8	1026.1	0.748	68.5	785.7
AJCC LN stage	0.739	268.2	1108.7	0.737	56.3	790
LNR stage	0.736	259.1	1122.6	0.741	58.7	785.5
LODDS stage	0.737	260.8	1112.4	0.725	52.3	794
Choi’s LN stage	0.742	271.2	1109.9	0.733	53.6	790.6
Chen’s LN stage	0.714	228.3	1152.4	0.721	50.7	793.5

*AIC* Akaike’s Information Criterion, *MMLN* modified metastatic lymph node, *AJCC* American Joint Committee on Cancer, *LNR* lymph node ratio, *LODDS* log odds of metastatic lymph nodes.

The MMLN staging system had the best discriminative ability (C-statistic = 0.748) and homogeneity (likelihood-ratio *χ*^2^ score = 68.5) in the CIAH validation cohort, but the LNR staging system performed somewhat better in terms of the AIC value (LNR vs. MMLN: AIC, 785.5 vs. 785.7). Survival curves for all six LN staging systems in the CIAH cohort are plotted in Fig. 2d−i. Although the MMLN staging system showed a difference in OS (*P* < 0.001), the MMLN2, MMLN3, and MMLN4 classifications failed to prognostically discriminate patients (log-rank test: MMLN2 vs. MMLN3, *P* = 0.361; MMLN3 vs. MMLN4, *P* = 0.320). Similarly, the last two or three subgroups within each of the other five staging systems also showed similar survival curves (all *P* > 0.05).

## Discussion

The prognostic values of PMLNs and EMLNs in patients treated with curative resection with at least a D2 lymphadenectomy for gastric cancer were studied. In multivariate analysis, both PMLNs and EMLNs showed prognostic values independent of the extent of gastrectomy, TLNs, and pT stage. We defined one EMLN as equivalent to ~2.6 PMLNs in terms of the degree of influence on prognosis. A modified numeric-based LN staging system, namely, the MMLN staging system was established by combining the prognostic weights of PMLNs and EMLNs. The new system provided good discriminative ability and homogeneity for data collected from two high-volume hospitals in the Eastern Asian countries.

LN status in gastric cancer is one of the most robust predictive variables of OS after gastrectomy and research has focused on defining an optimal LN-based staging system over the last decade.^23,32–36^ Even with the widely used AJCC TNM staging system, its recent editions have mainly optimised the LN staging cut-off values and number of subgroups.^37–40^ To our knowledge, the present study is the first to evaluate and demonstrate the differences in prognostic power between PMLNs and EMLNs in terms of their respective numbers. The MMLN staging system is established based on accurate information on the number of MLNs in each LN group, unlike the AJCC LN staging system, which uses the total number of MLNs only.

We used multiple indices, including Harrell’s C-index, likelihood-ratio *χ*^2^ score, and AIC to evaluate and compare the prognostic value of each LN staging system. Although all LN staging systems showed good predictive accuracy, the prognostic performance of the MMLN staging system was the best. The superiority of the MMLN staging system was demonstrated in survival analysis across the subgroups of the other five LN classifications (Fig. 2a−c and Table 3). The MMLN classification showed good discriminative ability within most subgroups of the other five LN staging systems and showed sufficient homogeneity within each MMLN-derived group across the other five LN staging systems. Furthermore, the results in the validation cohort strengthen our research. Both China and Japan are countries with a high incidence of gastric cancer in the world, but they have completely different epidemiological characteristics.^41^ Our study cohort also showed significant differences in clinicopathological characteristics, but the MMLN staging system in the CIAH cohort still showed good predictive power, indicating that our staging system has good universality. Therefore, in high-volume centres that routinely perform high-quality D2 lymphadenectomy, MMLN classification could be recommended for inclusion in the prognostic evaluation system. A D2 lymphadenectomy and assessment of the number of MLNs at each group are prerequisites for applying the MMLN staging system. Although the MMLN staging system requires knowledge of cut-off values and calculation formula, we believe that MMLN staging system is still simple and easy to use in clinical work.

Although the anatomic location of MLNs is no longer included in the TNM and JGCA staging systems, we considered it essential to improve the predictive accuracy of any LN staging system. Choi’s and Chen’s new LN staging systems also include the anatomic location of MLNs, but neither of them performed as well as the MMLN classification, and both performed worse than did the eighth edition AJCC LN staging system. Choi’s system divides the area of MLNs into LC, GC, and EP and focuses only on the presence of MLN in these three regions.^18^ This strategy ignores the importance of the number of MLNs, thereby weakening homogeneity. In addition, Choi’s system assumes equivalent prognostic effects of MLNs in these three areas. Further research should test if the prognosis of EP alone (equivalent to skip MLN), as in Choi’s N1 stage, is equivalent to that of LC and GC, and whether the prognosis of LC+EP and GC+EP (equivalent to JRSGC’s N2 group+) is the same as that of LC + GC (equivalent to JRSGC’s N1 group+) in Choi’s N2 stage. Such insights could improve the accuracy of Choi’s system. However, at present, these proposals remain controversial.^42–45^ Chen’s system is an LN staging system that combines the number and anatomic location of MLNs, but uses a cut-off value of overall MLNs to establish subgroups for the N1, N2, and N3 groups.^19^ However, in the present study, this system showed poor prognostic performance, suggesting using such a cut-off value is not accurate enough.

Recently proposed LN staging systems, such as LODDS and LNR, emphasise the importance of TLNs.^15,22,36,46^ However, in our study, when the MMLN staging system was included in multivariate analysis, TLN was excluded, suggesting that it is not an independent prognostic factor. In addition, high-quality D2 lymphadenectomy means adequate LNs dissection. In contrast, TLNs cannot be used to evaluate the extent of LNs dissection. Therefore, both LODDS and LNR staging systems did not perform better than did our MMLN staging system.

This study has several limitations. First, the extent of D2 lymphadenectomy was changed from a more extensive to the less extensive form between 2007 and 2015. To minimise the within-study heterogeneity likely to result from this change, we also included patients with D2 plus lymphadenectomy, according to the latest edition of Japanese gastric cancer treatment guidelines. Second, the MMLN staging is based on high-quality D2 lymphadenectomy along with the status of PMLNs and EMLNs. As such, it might not be suitable for patients recommended D1 lymphadenectomy or with fewer than 16 LNs retrieved. Further research is necessary to expand the applicability of the MMLN staging system to patients treated with D1-plus-lymphadenectomy. Third, although the MMLN staging system performed well in both PUCH and CIAH cohorts, it failed to discriminate some subgroups of patients within the CIAH cohort. This finding can be explained by a high proportion of patients with earlier-stage gastric cancer in the CIAH cohort. Although the distinct clinicopathological characteristics of the Japanese cohort strengthened the universality of MMLN staging system, the limited late-stage cases (AJCC pN3a + pN3b less than 10%) in the Japanese cohort also weakened the advantages of MMLN staging system in these stages. Using a larger cohort might help validate the prognostic performance of the MMLN staging system. Finally, this study included only Asian cohorts; future studies are required to validate the applicability to Western cohorts.

## Conclusion

To summarise, this study demonstrates the differences in prognostic power between PMLNs and EMLNs in terms of their respective numbers and establishes the MMLN staging system on the basis of the number of PMLNs and EMLNs. Comparisons with the eighth edition AJCC LN, LNR, LODDS, Choi’s, and Chen’s staging systems suggest better prognostic performance of the MMLN staging system. We recommend the MMLN staging system in patients treated with curative gastrectomy with at least D2 lymphadenectomy for prognostication.

## Supplementary information

Supplemental Figure and Table

## Data Availability

Data of the PUCH cohort are not shared, owing to the privacy or ethical restrictions. Data of the CIAH cohort are openly available through the Internet.
