# Supplementary material for: Development and validation of a novel staging system integrating the number and location of lymph nodes for gastric adenocarcinoma
Source: Br J Cancer. 2020 Dec 2;124(5):942–50. doi: 10.1038/s41416-020-01190-z (PMC7921685; doi:10.1038/s41416-020-01190-z)
Supplement: Supplementary file 1 — Supplemental Figure and Table [file 41416_2020_1190_MOESM1_ESM.pdf]

British Journal of Cancer

Development and validation of a novel staging system integrating the number and location of lymph nodes for gastric adenocarcinoma

Ziyu Li[1,*], Xiaolong Wu[2,*], Xiangyu Gao[1,*], Fei Shan[1], Xiangji Ying[1], Yan Zhang[1], Jiafu Ji[1,#]

[1]Gastrointestinal Cancer Center, Key Laboratory of Carcinogenesis and Translational Research (Ministry of Education), Peking University Cancer Hospital and Institute

[2]Gastrointestinal Cancer Center, Key Laboratory of Carcinogenesis and Translational Research (Ministry of Education), Peking University Cancer Hospital and Institute, E-mail: kimi112358wxl@hotmail.com

[*]These authors have contributed equally to this work

# Corresponding to:

Jiafu Ji

Gastrointestinal Cancer Center, Key Laboratory of Carcinogenesis and Translational Research (Ministry of Education), Peking University Cancer Hospital and Institute, No. 52 Fu-Cheng Road, Hai-Dian District, Beijing 100142, People’s Republic of China

Tel: +86 13801096270

E-mail: jijiafu@hsc.pku.edu.cn

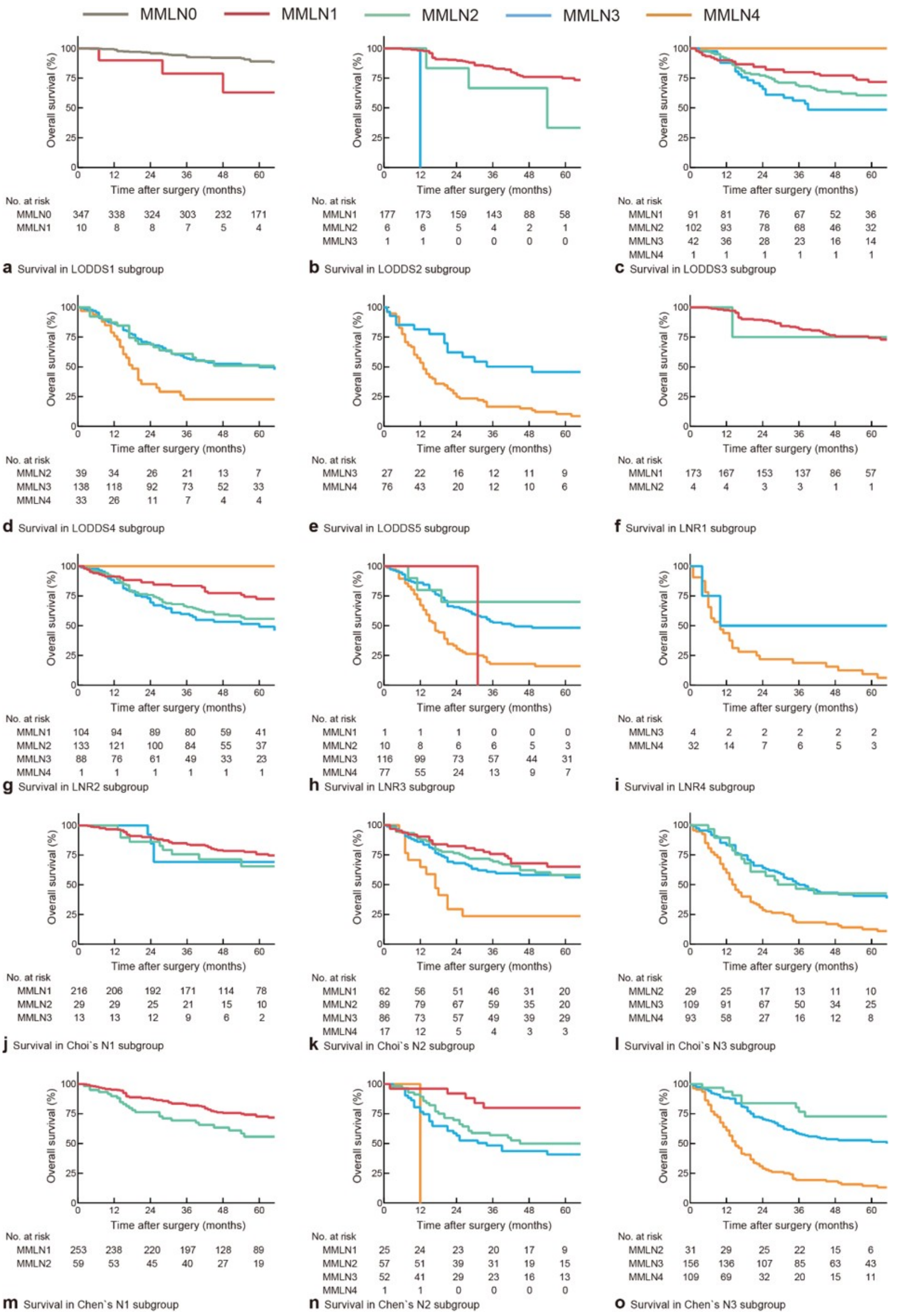

Fig. S1 Kaplan-Meier curves for overall survival stratified by the MMLN classification within LODDS, LNR, Choi`s, Chen`s staging systems in the PUCH cohort. **a** LODDS1 subgroup, **b** LODDS2 subgroup, **c** LODDS3 subgroup, **d** LODDS4 subgroup, **e** LODDS5 subgroup, **f** LNR1 subgroup, **g** LNR2 subgroup, **h** LNR3 subgroup, **i** LNR4 subgroup, **j** Choi`s N1 subgroup, **k** Choi`s N2 subgroup, **l** Choi`s N3 subgroup, **m** Chen`s N1 subgroup, **n** Chen`s N2 subgroup, **o** Chen`s N3 subgroup. **a** *P* = 0.053, **b** *P* < 0.001, **c** *P* = 0.014, **d** *P* = 0.001, **e** *P* < 0.001, **f** *P* = 0.784, **g** *P* = 0.001, **h** *P* < 0.001, **i** *P* =0.132, **j** *P* = 0.231, **k** *P* = 0.001, **l** *P* < 0.001, **m** *P* = 0.013, **n** *P* = 0003, **o** *P* < 0.001 (log rank test). MMLN, modified metastatic lymph node; LNR, lymph node ratio; LODDS, log odds of metastatic lymph nodes.

Table S1 MMLN subgroups according to the number of MMLN with top 10 maximum C index using enumeration method.

MMLN subgroups				C index
MMLN1	MMLN2	MMLN3	MMLN4	
1-4	5-8	9-20	>20	0.75131
1-4	5-9	10-20	>20	0.75127
1-4	5-10	11-20	>20	0.75122
1-4	5-8	9-18	>18	0.75110
1-4	5-9	10-18	>18	0.75056
1-4	5-8	9-19	>19	0.75057
1-4	5-10	11-18	>18	0.75042
1-4	5-9	10-19	>19	0.74979
1-4	5-12	13-20	>20	0.74972
1-4	5-10	11-19	>19	0.74966

MMLN, modified metastatic lymph node.

Table S2 Univariate and Multivariate analysis using Cox proportional hazard regression model in validation cohort.

Variable	Univariate			Multivariate		
	HR	95% CI	*P* value	HR	95% CI	*P* value
Age (years)						
≤60 (reference)	1.00		<0.001	1.00		0.002
>60	2.46	1.47-4.10		2.28	1.36-3.83	
Sex						
Male(reference)	1.00		0.051			
Female	0.55	0.30-1.00				
Tumor size (cm)						
≤5 (reference)	1.00		<0.001			
>5	3.56	2.17-5.84				
tumor location						
U (reference)	1.00		0.971			
M	0.45	0.25-0.83				
L	0.73	0.40-1.35				
Overlapping lesion	1.78	0.52-6.07				
extent of gastrectomy						
distal (reference)	1.00		<0.001			
total	2.91	1.82-4.66				
differentiation						
differentiated (reference)	1.00		0.567			
undifferentiated	1.16	0.71-1.89				
histological type						
Adenocarcinoma (reference)	1.00		0.225			
signet ring cell carcinoma	0.65	0.37-1.12				
mucinous adenocarcinoma	1.12	0.27-4.60				
pT stage						
T1 (reference)	1.00		<0.001	1.00		<0.001
T2	4.28	1.55-11.83		2.74	1.21-9.80	
T3	10.31	4.32-24.62		2.45	2.41-17.21	
T4a	17.38	7.61-39.68		4.11	3.37-23.90	
T4b	14.71	3.77-57.41		6.29	2.72-48.41	
TLNs						
≤30 (reference)	1.00		0.043			
>30	1.90	1.02-3.54				
MMLNs						
MMLN0 (reference)	1.00		<0.001	1.00		<0.001
MMLN1	3.41	1.75-6.62		1.28	0.61-2.69	
MMLN2	8.23	4.00-16.95		2.54	1.11-5.83	
MMLN3	10.80	5.21-22.38		3.20	1.38-7.45	
MMLN4	18.00	5.88-55.07		3.69	1.10-12.38	

TLN, total number of lymph nodes retrieved; MMLN, modified metastatic lymph node.
